# Rationing is a reality in rural physiotherapy: a qualitative exploration of service level decision-making

**DOI:** 10.1186/s12913-015-0786-3

**Published:** 2015-03-27

**Authors:** Robyn Adams, Anne Jones, Sophie Lefmann, Lorraine Sheppard

**Affiliations:** 1 Discipline of Physiotherapy, College of Healthcare Sciences, James Cook University, 1 James Cook Dr, Douglas QLD 4811, Townsville, Australia; 2 Discipline of Physiotherapy, University of South Australia, Adelaide, Australia

**Keywords:** Physiotherapy, Rural, Regional, Health Services, Prioritization, Rationing, Decision-making

## Abstract

**Background:**

Deciding what health services are provided is a key consideration in delivering appropriate and accessible health care for rural and remote populations. Despite residents of rural communities experiencing poorer health outcomes and exhibiting higher health need, workforce shortages and maldistribution mean that rural communities do not have access to the range of services available in metropolitan centres. Where demand exceeds available resources, decisions about resource allocation are required.

**Methods:**

A qualitative approach enabled the researchers to explore participant perspectives about decisions informing rural physiotherapy service provision. Stakeholder perspectives were obtained through surveys and in-depth interviews. A system theory-case study heuristic provided a framework for exploration across sites within the investigation area: a large area of one Australian state with a mix of rural, regional and remote communities.

**Results:**

Thirty-nine surveys were received from participants in eleven communities. Nineteen in-depth interviews were conducted with physiotherapist and key decision-makers. Increasing demand, organisational priorities, fiscal austerity measures and workforce challenges were identified as factors influencing both decision-making and service provision. Rationing of physiotherapy services was common to all sites of this study. Rationing of services, more commonly expressed as service prioritisation, was more evident in responses of public sector physiotherapy participants compared to private physiotherapists. However, private physiotherapists in rural areas reported capacity limits, including expertise, space and affordability that constrained service provision.

**Conclusions:**

The imbalance between increasing service demands and limited physiotherapy capacity meant making choices was inevitable. Decreased community access to local physiotherapy services and increased workforce stress, a key determinant of retention, are two results of such choices or decisions. Decreased access was particularly evident for adults and children requiring neurological rehabilitation and for people requiring post-acute physiotherapy. It should not be presumed that rural private physiotherapy providers will cover service gaps that may emerge from changes to public sector service provision. Clinician preference combines with capacity limits and the imperative of financial viability to negate such assumptions. This study provides insight into rural physiotherapy service provision not usually evident and can be used to inform health service planning and decision-making and education of current and future rural physiotherapists.

## Background

The challenge of delivering health services equitably to Australia’s rural population is exacerbated by health workforce maldistribution and fewer services in rural areas. Australia’s 23 million people are spread across 7.6 million square kilometres and it is widely accepted that 30% of the population live outside major metropolitan areas, in areas broadly described as rural Australia. There are many definitions and classifications used to describe or differentiate regional, rural and remote settings [1,2]. The Australian Standard Geographical Classification Remoteness Areas (ASGC-RA) is recommended by the Australian Institute of Health and Welfare (AIHW) [3]. Other Australian classifications include the Access/Remoteness Index for Australia (ARIA) and Rural, Remote and Metropolitan Areas Classification (RRMA). The classifications are based on factors such as distance to service centres, population size or density. So while ninety percent of Australians in 2011 lived in urban areas (defined as cities or towns of more than 1,000 people), many were small communities in rural areas [4].

Health workforce shortages and geographic maldistribution compound the challenge of delivering equitable health services beyond major cities. An estimated 80% of physiotherapists, for example, worked in major cities in 2012 ([5], p52). Despite residents of rural and remote communities experiencing poorer health outcomes and exhibiting higher health need, many rural and remote communities do not have access to the range of health services as large urban centres [6-9]. The challenge then becomes one of deciding what health services should be provided, where and to whom.

The impossibility of providing everything to everyone means making choices is inevitable. Where there is a demand-resource imbalance, decisions about resource allocation are required. Prioritisation, alternatively referred to as resource allocation or rationing, occurs at all levels of the health care system where demand exceeds available resources [10-13]. Rationing involves addressing questions such as: what treatments or healthcare services should be provided? How should these services be distributed amidst budgetary constraints? Who decides? How? Based on which criteria? ([14], p.63). Deciding what health services are provided is a key consideration in delivering appropriate and accessible health care for rural and remote populations [8].

Physiotherapists are autonomous health care professionals and form an important part of the rural and remote health workforce [15,16]. Physiotherapists *play a key part in the acute care and rehabilitation of their clients and the promotion of health in their communities* [17]. Provision of physiotherapy services in rural communities is not well described, however physiotherapy workforce shortages and geographic maldistribution infer fewer services compared to metropolitan settings. Rural physiotherapy literature describes workforce demographics and distribution, areas of work and workforce stressors [16,18-20]. Workforce stressors described in regional settings, including caseload quantity, increased activity, patient complexity and constant excessive workload, are reflective of service provision challenges [20]. When combined with broader health system challenges such as increasing chronic disease, an ageing population and fiscal constraints, they also form the stimuli for prioritisation or rationing of physiotherapy service provision.

In contrast to clinical decision-making, which is well described in the physiotherapy literature, there is relatively little describing physiotherapy decision-making at a service level [21-28]. An emerging literature informing physiotherapy decision-making about service provision includes prioritisation of patient populations and physiotherapy and allied health caseload measurement and management [29-34]. This literature is indicative of the demand-resource imbalance that requires rural physiotherapists to make decisions about service prioritisation or rationing of services. How decisions are made about which physiotherapy services are provided in rural and regional Australia, is not evident in the rural physiotherapy literature. This paper explores decision-making about physiotherapy service provision in eleven rural and regional communities against a context of health care rationing. Definitions of rationing and common criteria are used to frame participant responses and guide discussion.

### Rationing

Rationing, defined as *the distribution of resources between programmes and persons in competition* takes place at all organisational levels ([14], p.63). Three levels commonly referred to in health care are macro, meso and micro levels (Table 1), with decisions at higher macro levels often constraining lower level options [10,11,14,35,36]. In this study the term ‘macro’ is used to refer to health related factors that occur at a national or state level, ‘meso’ is used to refer to factors located at a regional /facility level and ‘micro’ those that occur at the physiotherapy service level.

**Table 1:** **Organisational decision-making levels**

Macro	Meso	Micro
the national or regional level, where the healthcare budget is decided…includes decisions regarding increases or reductions in spending, or financing of particular programmes.	local level (regional or hospital), where resources are allocated to different functions and local authorities make decisions about local priorities.	the care level, where healthcare professionals make decisions about who, how, when, where and how to care for patients.
…represents the key constraint within which further divisions of funds between regions and local health providers.	…choices may involve the priorities attached to treatment services versus preventative medicine; particular patient groups, or certain hospital services.	…the question of professional prerogative can be limited by constraints from above.

Adapted from Putoto and Pegoraro 2011 pp64-5.

Rationing may be explicit, with decisions and their rationale made open and transparent or implicit, where neither the decisions nor the reasons are clearly expressed [35]. Explicit and implicit rationing are well discussed in relation to macro level health service decisions, for example in the UK, New Zealand, Sweden and Oregon (Table 2) and in the medical literature [14,37]. Mechanic defined the terms as follows:

*Explicit rationing* refers to decisions made by an administrative authority as to the amounts and types of resources to be made available, eligible populations, and specific rules for allocation.

*Implicit rationing*, in contrast, refers to discretionary decisions made by managers, professionals, and other health personnel functioning within a fixed budgetary allowance ([37], p87).

**Table 2:** **Macro rationing strategies: International examples**

Oregon	Netherlands	Sweden	New Zealand	Great Britain
Explicit list of funded treatments:	4 Criteria:	3 Principles:	Confirmed essential services and developed guidelines for high cost and high volume services.	Delegated priority setting to local authorities with national agencies undertaking treatment evaluation and service performance.
(Originally *565 of the 696 listed treatments*).	-Necessity	-Human dignity		
	-Efficacy	-Need and solidarity		
	-Efficiency			
	-Individual	-Cost/efficiency.		
	responsibility.			

After Putoto and Pegoraro 2011 p73-75.

While there has been a shift from implicit toward explicit rationing at a macro level, the presence of explicit rationing decisions do not fully resolve the dilemmas facing decision makers [14,38]. The changes at a macro level have been accompanied by the strategies at a meso and micro level. Evidenced based guidelines are an example of micro level strategies which individual clinicians must interpret and apply in the clinical setting [38]. It has been suggested that making choices in health care involves making judgements about the relative priority of different objectives and services ([38], p.164). Such judgements, or ability to make considered decisions or come to sensible conclusions, requires a level of self-efficacy and competence in decision-making ([39] p.94). The challenge of making decisions when faced with potential incompatibility between service objectives and the needs of a specific patient or client group, is one faced by many clinicians at the micro level. For example, the potential incompatibility between efficiency and equity means that conflict between objectives and trade-offs are a likely consequence. An understanding of individual and community preferences and values can inform these choices.

The discussion below on criteria, particularly distributive criteria, provides an added dimension to decision-making about the allocation of resources. Clarifying values that guide decisions about health care rationing at a macro level can assist decision-making about rationing or prioritisation of services at lower levels. However, there still remain challenges for the day to day application of rationing decisions at health service (meso) level and at the level of the clinician (micro level).

In Australia, a set of principles underpin the design of Australia’s future health system. The National Health and Hospitals Reform Commission developed these in two functional categories [40]. Firstly, service design principles (generally what citizens and potential patients want from the system 1–8) and secondly, governance principles (generally how the health system should work 9–15) (Table 3) [40].

**Table 3:** **National Health and Hospital Reform Commission design principles**

Service design principles	Governance principles
1. People and family centred	9. Taking the long term view
2. Equity	10. Safety and quality
3. Shared responsibility	11. Transparency and accountability
4. Strengthening prevention and wellness	12. Public voice
5. Comprehensive	13. A respectful and ethical system
6. Value for money	14. Responsible spending on health, and
7. Providing for future generations	15. A culture of reflective improvement and innovation
8. Recognise broader environmental influences which shape our health	

### Criteria

**Figure 1:**
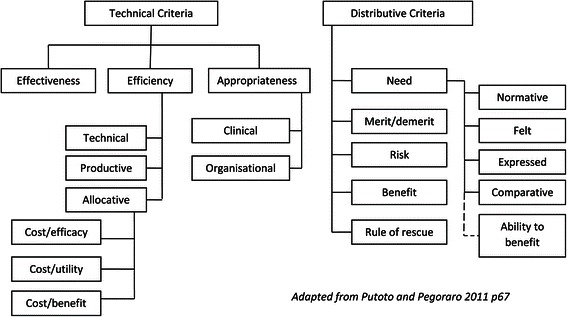
**Technical and distributive criteria.**

Criteria reflecting prevalent society values can assist to establish priorities or make choices at all organisational levels. Criteria are used to inform health policy and planning at macro and meso levels and are reflected in micro level decision-making of clinical health professionals. Criteria have been classified as technical or distributive [14]. Technical criteria refer to qualities that services must possess and have been suggested to be a prerequisite in any selection of priorities [14] [p67]. Whilst they can exclude interventions, they are not sufficient in themselves to establish how many, or which intervention to provide and to whom. Distributive criteria are a set of principles that establish an order of priority in the allocation of healthcare resources. They do not address the question of what must be guaranteed to individuals and society at large, but do help establish order of priorities in the choice between different patients or patient groups (Figure 1) [14].

Rationing, perhaps a more emotive term than priority setting, is often not deliberate or conscious process ([14], p66). Where rationing occurs at a micro level, it is often a means for professionals to cope with budgetary or other pressures and reflects the challenge for the day to day application of rationing decisions. Consideration of both technical and distributive criteria and a range of rationing methods, such as selection, denial, deflection and delay (Table 4) are strategies health professionals can adopt to aid their decision-making.

**Table 4:** **Methods of rationing**

Selection	Using this method, recipients of care are selected on the basis of clinical benefit they will obtain, or the amount of time required to treat them.
*Denial*	This method involves the exclusion of certain patient populations because they are deemed unworthy, or because their needs are not seen as sufficiently important.
*Deflection*	This involves referring patients to other institutions. It is a form of rationing when a patient’s needs can be met by other health or social services.
*Deterrence*	This involves deterring patients from accessing healthcare by the imposition of complex logistical/administrative requirements, such as inconvenient opening times, incomprehensible paperwork, and unhelpful staff. This type of rationing tends to disadvantage less educated and more vulnerable people.
*Delay*	This method includes the use of waiting lists. It is the most recognised form of implicit rationing in healthcare, and discourages patients from accessing health services.
*Dilution*:	In this situation access to services is not denied, but the provision of services is reduced, such as the frequency of home visits.
*Interruption*	This is the premature termination of a service or a treatment based on a maximum time limit for a given treatment, such as premature discharge from hospital or case closure.

*After Putoto and Pegoraro 2011* p66.

This study’s primary aim was to obtain participant perspectives on decision-making about the provision of physiotherapy services in selected rural and regional communities. Considering their perspectives within a context of decision-making criteria and health care rationing enabled placement of perspectives within the broader health system context.

## Methods

An iterative data collection process incorporating both quantitative and qualitative components assisted initial identification of issues and subsequent exploration of stakeholder perspectives of identified issues. The prioritised qualitative component enabled the researchers to explore participants’ thoughts and perceptions. An interpretivist approach within a qualitative research paradigm supported understanding of stakeholder perspectives [41]. Stratified purposive sampling permitted exploration of subgroups of interest [42]. The subgroups include physiotherapists, their colleagues, managers and key decision-makers and consumers. The investigation site was a large area of one Australian state with a mix of regional, rural and remote communities.

Data collection commenced with a survey distributed to all public sector physiotherapists working in the geographical area of the study. Public sector physiotherapists were prioritised as a greater reliance on the public sector for the provision of allied health services, including physiotherapy, has been reported in rural and remote regions of Australia [43]. Information obtained from the physiotherapist survey then informed the selection of case sites and issues for exploration in the qualitative phase of the study. A matrix, reflecting two key aspects of the research, assisted case selection and subsequent participant sampling. Key aspects were rurality (regional, rural or remote) and the number of physiotherapists working in the same service. The researchers’ experience in rural physiotherapy, suggested that these two aspects may assist in the differentiation of sites for exploration of physiotherapy service level decision-making. Exploring decision-making within a systems theory-case study heuristic enabled consideration of location and system level influences [44].

Consenting physiotherapists at each case site then distributed surveys to colleagues, managers and consumers relevant to their service. The physiotherapists were invited to participate in a semi structured, in-depth interview. They were also asked to provide suggestions of key decision makers who could be invited to participate in interviews. Surveys were sent to private physiotherapy practitioners in each case site by the researcher. Private physiotherapy practitioners were identified through listings in the yellow pages phone directory and were approached in order of listing.

Manual and electronic recording of data through the use Microsoft Excel Spread sheets and NVivo version 10 were used to organise the data. An iterative approach was used to guide the data analysis [45]. The levels of analyses included: ongoing preliminary analysis to critique data as it came in; thematic analysis to develop tentative themes; coding, including the generation of themes and concepts to develop codes, which were then used to frame account for the remaining data [45]. The research design included data collection from multiple sources to enable triangulation of data and constant comparison. Interviews were audio taped and transcribed verbatim with full transcripts and researcher summaries returned to participants for verification and the opportunity to provide comments or corrections. One third of the interviews (7/19) were coded by a second coder to verify themes. An auditable trail of evidence was maintained throughout the conduct of the research to further add to the credibility of the findings [45-47]. Written consent was obtained prior to completion of surveys or interviews. Data collection occurred from January to September 2012. Ethics approval was obtained from the Human Research Ethics Committee of both James Cook University (approval number H3799) and the health services of the study.

## Results

A total of 39 surveys were received and 19 interviews were conducted. Survey responses were received from 16 public sector physiotherapists (29.6% response rate) from 11 of the 25 (44%) facilities identified as providing physiotherapy services in the investigation area. Six case types relevant to the exploration of rural physiotherapy service provision emerged: rural ≤ 1; rural 2–3; rural 4–10; rural-remote 4–10; regional 4–10 and regional > 10. The cases are named firstly by participant perception of rurality (regional, rural or remote) and secondly, by the number of public sector physiotherapists expressed in fulltime equivalents. Examples include a rural community with one fulltime and one part time physiotherapist (rural 2–3) or a regional centre with more than 10 public sector physiotherapists (regional > 10). Following distribution of subsequent stakeholder surveys within the case sites, 13 colleague-manager responses, 6 consumer responses and 5 private physiotherapy practitioner responses were received. A total of 39 surveys remained after one consumer participant was identified as ineligible (under 18 years of age) (Table 5).

**Table 5:** **Participants**

Physio FTE		≤1	2–3	4–10	>10
**Remote**	Surveys				
	Interviews				
**Rural- Remote**	Surveys			4P, 4CL	
	*Interviews*			*1DM, 1P, 1PP, 1CL*	
**Rural**	Surveys	4P, 3CL, 1CN	2P, 1PP 2CL, 2CN	1P, 1PP, 1CL	
	*Interviews*	*2P, 1DM*	*1P, 1PP*	*1DM, 1P, 1PP*	
**Regional**	Surveys			4P, 2PP, 1CN	1P, 1PP, 3 CL, 1CN
	*Interviews*			*2P, 1PP*	*1DM, 2P, 1PP*

*CL: colleague; CN: Consumer; DM: decision maker; P: public physiotherapist; PP: private physiotherapist.*

Site visits occurred in September 2012 when the principal researcher conducted face to face interviews with participants. Nineteen participants were interviewed: 9 public sector physiotherapists; 5 private practitioner physiotherapists; 4 key decision-makers and one colleague. Interviews time ranged from 45 minutes to 120 minutes. Interview participant perceptions have been coded alpha-numerically in the following groups: public physiotherapists (A); private physiotherapists (B); colleagues (C); and decision-makers (D). Open ended survey questions and interview data provided perspectives on how decisions were made about service provision.

### Surveys

Survey responses from physiotherapists, colleagues and managers to questions about how decisions were made about service provision and what factors influenced those decisions were analysed to identify key issues. Responses from physiotherapists revealed variable mechanisms and considerations about how decisions are made about which physiotherapy services are provided. Independent decision-making by private physiotherapists contrasted with public sector physiotherapist responses which reflected the broader influence of both health service requirements and the community. Workload management, waiting lists and service prioritisation within the public sector contrasted to private physiotherapists’ responses of expertise, clinician preference and affordability. Whilst no specific rationing strategy was framed in terms described by Putoto and Pegoraro (Table 4), waiting lists and service prioritisation were examples of delay and selection [14].

Colleague and manager survey responses revealed variable levels of knowledge of how decisions were made about which physiotherapy services are provided. Options included service decisions made by physiotherapist independently or after consultation with colleagues and managers; in response to directives and institutional or local demands. Considerations included staffing levels and expertise, workload demands and agreed core business.

Consumer responses were to the survey question about how the physiotherapy service could be improved. It was not assumed that consumers would be decision makers about service provision, but rather be drivers of service demand. Consumer responses (CN) included:

*Let there be more* [CN 1].

*More physios-though very happy with my service* [CN 2].

*Lymphoedema physios give help with Laser, massage, garments, advice etc. The physios in [this town] work very hard. Not enough of them!!* [CN 4].

*An increase in funds available* [CN 5].

*More related services under one roof eg massage, scanning* [CN 6].

### Interviews

In depth interviews then enabled exploration of issues identified in the surveys. Decision-making about service provision was considered within organisational levels and rationing strategies used. Responses revealed macro and meso level decision-making influences which then framed decision-making at a service or micro level. Decisions impacting the provision of physiotherapy services in the rural and regional settings fell under two broad areas: health reforms or funding decisions. These two areas provide the organisational priorities on resource allocation that inform service level decision-making (SLDM).

Meso level decisions identified by participants include those made at a regional or facility level. These decisions influenced the organisation and funding of services including service directives, priorities, funding and staffing levels. Examples of meso level decisions noted by participants include centralisation of services (from remote and rural areas to regional centres), budget and staff cuts, local implementation of national funded programs (eg subacute care), organisational priorities and performance indicators (length of stay, waiting list management, revenue targets).

Decisions at meso and macro levels then provide the framework for decisions about service provision at a micro level within clinical department or units. At this level, multiple methods of rationing or priority setting were evident in responses of physiotherapy participants. Responses reflected a language of priorities (*you spread yourself pretty thin; you do prioritise, you have to* [A4])*,* rather than rationing, with no reference to specific methods of rationing. However, many of the strategies or processes described by participants (Table 6) align with the rationing methods described by Putoto and Pegoraro [14]. Rationing decisions were more apparent in public sector physiotherapy responses. Private physiotherapist responses revealed issues of capacity and self-direction.

**Table 6:** **Methods of rationing used by physiotherapy participants**

Selection	**A3:***Priority one is the ICU sort of work: your very early stage orthopaedic patients, your day one gut surgeries, day one strokes, that sort of stuff and then right down the bottom of the list is mobility aid assessments, mobility reviews.* [ED is] *an artificial priority …it wouldn’t come up as a first thing that we would do, but because of the funding, it’s one that’s going to be maintained regardless of what is happening anywhere else.*
	**A3:** *Sub-acute [care program]: at the moment, their core area is fractures over 65, so they do ortho-geriatrics.*
	**A9:***I’ve only got a certain amount of time…it’s an awful situation to have, but this is the one I have to spend more time with …to know where am I going to get the best outcome … you can’t treat them equally and that’s always been a frustration I think*.
	**A1:** *Yes acuity and being in hospital and getting people out of hospital because anybody in health that is looking at dollars, looks at length of stay and it’s the only thing that counts.*
*Denial*	**A9:** [when we had less than half our staff] *we had set wards* [as the priority] *and we closed outpatients.*
	**A1:** [staff cuts] *severely curtailed our ability to provide outpatient services. We’ve had to basically can [cease] any outpatient rehabilitation service.*
	**A1:***When* [the paediatric physio leaves], *there will be a gap because I can’t pick up* [paediatric] *neuro type or the disability, I can’t do it. I can’t do everything to that level.*
*Deflection*	**A3:** [Physio X’s] *job is to try and help flow them out to peripheral [hospitals], even if they’re from here they might go out to [a peripheral rural hospital] where staff there can continue their exercises and help them not weight-bear and then once they’re able to weight-bear, then they are appropriately brought back to rehab.*
	**A3:***Three out of four of our patients come from outside of* [this Regional city] *so that’s the other thing…what’s available at the other end very much determines how easily we can move people on.*
	**A9:***That’s right; so sometimes people need to be transferred to* [Metropolitan centres] *for anything more complicated.*
	**A6:** *Well the in-patients, we have no influence who comes in as an in-patient. So our influence is then on who we send out the community, who we send to rehab…*
	**A3:***Sometimes we are sending referrals out into the ether knowing that the town that that knee replacement patient is from, doesn’t have a physio, and there’s nothing that I can do except send that referral through, knowing there will be receipt at the other end and registered as a need, but I can’t do anything else*.
	**A6:** *Out-patient wise, we have quite a lot of private practices within the area and they’re able to take all third parties and anyone with private insurance or if our waiting list is too long, we suggest other people go along and at least get initial treatment…*
	**A3:***We channel those* [private or compensable patients] *to private, but there’s a lot of demand*
	**B5:***they might come through* [to the practice] *and say “I've had a stroke” and I’ll think I’ll be more than happy to look at you…but I look at them, assess them and think, I really don’t have the services here, or the rehab equipment here to do that for them, yeah I refer them off* [to the public service].
*Deterrence*	**B5:***I think Case Managers put so much strain and stress on you, you’re trying to get someone better and they’re declining treatment and those sorts of things. …you’re getting someone back to work and then all of a sudden they stop the services and then the client goes backwards and returns to being off work*…
*Delay*	**A3:** *Certainly there’d be waiting lists for Paeds…and general out-patients definitely.*
	**A9:** *Okay so with our acute that’s under 2 weeks…so they usually get them in within a couple of days; and certainly if anything comes across from ED …that’s on the spot stuff, and then we have 2 to 8 weeks – so your sub-acute and they probably take 2 weeks to get in.... and then greater than 8 weeks we usually go a month.*
	**A7:** *I must provide the care for the acute inpatients and ED services – that is my core. I can do that and then there are the outpatients that can be sorted into waiting lists.*
*Dilution*	**A3:***Absolutely*, [the post-acute service] *are able to see people for six weeks post op or post hospital stay but they’re limited to 25 kms from here. So if you live in* [this regional city] *you get a great deal, you get six weeks of home visits essentially but as soon as you’re one metre out of that 25 kms, all you have is musculoskeletal outpatient* [physiotherapy at] *your local facility*.
*Interruption*	**A9:** *Out by day 5 or 6*
	**A3:** [still send to a town that, at the moment doesn’t have a physio] *Of course we do, but I can’t send them home with the same level of input as if they were local. There are lots of towns with no private practice …or because there’s no one at* [the rural hospital] *for three weeks. So you kind of just have to move them on anyway and the ability to bring them back, I can’t do that, we’re restricted.*

Examples of criteria used to inform service provision were evident in a number of participant responses, particularly technical criteria (Table 7). One participant (A5), who worked part time and at times was the only physiotherapist, highlighted a number of ‘distributive criteria’ when discussing service priority decisions. This participant considered the evidence on effectiveness and expected outcome or benefit and made a judgement about the relative priorities of the competing demands on her time. In doing so, this participant demonstrated an understanding of individual and community needs. At times, contrary to the expected prioritisation of inpatients, this participant prioritised paediatric outpatients.

**Table 7:** **Physiotherapy examples of applications of technical and distributive criteria**

**Technical Criteria**	
Effectiveness	**D1:** we’ve very much put that on current practice and research.
	**A2:** so we haven’t done any sort of study on that and I think that they’re hand in hand, we can only grow the service if we’ve got the evidence of best practice that we can then put to people who have money and purse strings… but we will never be able to, it would take a real reshuffle of how we do our work as two part timers to actually see how we could incorporate that.
Efficiency	**D2:** Look the biggest shift I think in Physiotherapy and Occupational Therapy is going to be in the ABF environment, and that’s going to be around the efficiencies and comparison between our services across our site, to look at the time of the intervention of Physiotherapy to particular diagnostic related groups based on the funding received.
	**D1:** we’re always trying to work out where we’re getting our best bang for our buck and where the resources are best spent.
Appropriateness	**D1:** I think it’s about the services the hospital offers and looking at our staffing, where we think we can make the most impact. So we’ve had a lot of say over where we provide the services.
**Distributive Criteria**	
Need	**D1:** But whether that’s a values based thing too, rather than just choosing, it’s hard to call.
	**A9:** So we look at the needs in our community and try and skill up with what we can do that makes it easier for them.
Merit/Demerit	**A5:** Oh constant friction – self friction – so it’s a judgement call, it’s not a right or wrong and other people may prioritise differently. I think it’s so simplistic to say that they’re acute patients…that they are inpatients so they deserve to be treated…So anyone that I don’t need to see I try not to… I certainly see people who are deteriorating.
Risk	**A5:** “Do they really need to see me?” “Yes I can see them as an out-patient”, and there is some pressure from them [ward nurses] but I can go back to the doctor and say this is why I haven’t see your patient; I do see its importance, but I’ve had to prioritise and I don’t get much problem with that, but the nursing staff I will get more just “Oh we never see the physio”.
Benefit	**A5:** but someone with just a chest infection to me, I’m going to have a limited evidence based effect on this person whereas someone with a serial cast after botox that’s where I need to prioritise.
Rule of rescue	**A3:** Priority one is the ICU sort of work.
	**A9:** and certainly if anything comes across from ED that’s on the spot stuff.

## Discussion

Rationing or prioritising physiotherapy service provision was common to all sites of this study, particularly the public hospital based services. The imbalance between increasing consumer need and organisational demands on the one hand, and constrained physiotherapy service capacity on the other, meant making choices was inevitable. The necessity of making such choices, while not ‘liked’ appeared to be seen as a necessary part of public sector physiotherapy service provision. Making choices about physiotherapy service provision involved making judgements about the relative priority of the different demands placed upon the physiotherapy service. Resignation, frustration and recognition of inequity, evident in physiotherapy responses, were accompanied at times by pragmatic acceptance, dismay or underlying anger. Two key issues combine to produce this increasing impost on micro level clinicians to make rationing decisions. The first is the relative invisibility of the range and scope of physiotherapy services to macro and meso level decision makers. The second is the devolvement of decision-making to the micro level by regional and facility levels. The constrained capacity to respond to increasing organisational activity, patient complexity and acuity echo the workforce stressors described in regional settings [20].

**Figure 2:**
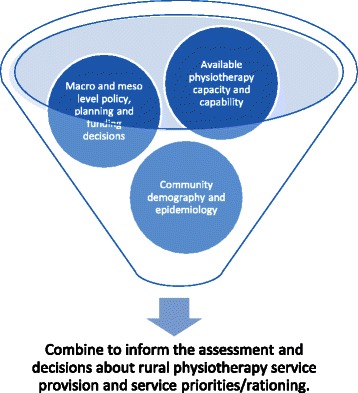
**Factors informing physiotherapy service priorities.**

Rationing physiotherapy services was more evident in the public sector. Private sector physiotherapist reported greater decision-making autonomy over their scope of practice, which was guided by their business model and aligned to their areas of expertise. The need to ration or prioritise public sector physiotherapy services appeared to stem from a number of reasons. Increased community demand and organisational activity, targeted funding and service priorities combine with shortages in both available physiotherapy workforce and funded positions to necessitate rationing of services (Figure 2).

Workforce shortages in rural areas, including a shortage of physiotherapists are well described, however the shortage of positions, though mentioned, is less evident in the peer reviewed literature [5,18,20,43,48,49]. The reductions in the number of funded positions, described by participants in this study, further exacerbated challenges in service provision capacity. Subsequent implications of rationing physiotherapy services in rural areas are numerous. Time restrictions resulting from decreasing length of stay and early discharge can then compromise optimal service delivery and safe, effective discharge. Also important in rural and regional communities is the reduced access to physiotherapy services such as outpatient rehabilitation for both neurological conditions and orthopaedic procedures. Despite targeted funding for subacute care, variable access to subacute rehabilitation services was reported in many communities. Limited access to specialist paediatric physiotherapy for children with complex neurological or developmental conditions was also common across sites of this study. Decision-makers should be cautious about assuming private physiotherapy providers will cover service gaps that may emerge from changes to public sector service provision. Rural private physiotherapy practices have very real capacity limits; including the range of expertise, space and affordability. These combine with clinician preference and financial viability to negate such assumptions.

Conflict with professional and personal values was one implication of service rationing on individual physiotherapists charged with the dual dilemma of service provision within public sector budget constraints. Rural physiotherapy service provision required physiotherapists to make judgements about the relative priority of the competing demands on their service. Numerous examples of SLDM by physiotherapists were made in response to factors stemming from system levels beyond their sphere of influence. Macro level decisions, such as the introduction of ABF, national performance targets and funding new programs, shifted organisational priorities. While responsiveness to the new priorities was expected, there was variable higher level direction about what services could be reduced or not be provided at all where demand exceeded available resources. Examples described by participants included new priorities following the introduction of new services (Emergency Department physiotherapy and subacute care). Similarly, decisions made at the regional or facility level, such as the reduction in funded positions or the delays in recruitment approvals, required physiotherapists to review what services could be maintained and which aspect of service provision would not be continued. These higher level decisions, often made without explicit directives about service reductions, effectively devolved the decision of rationing service provision to physiotherapists at a micro level. The consequences were expressed in terms of patients (reduced access to service and inability to provide follow up treatment) and the physiotherapists themselves (frustration and friction).

Limited communication and consultation about decisions by higher level decision makers to reduce service capacity or indeed add new services without additional physiotherapy resources compounded the conflict between service, professional and personal values. Perceived lack of autonomy is a key influencing factor in the retention of health rural health professionals [50]. Findings in this study of escalating workloads and a sense of being overwhelmed are consistent with key issues influencing retention of health rural health professionals [50]. Such negative work factors more significantly influence retention where there is no personal connection to the community [50,51]. The decision to stay then depends on factors such as personal resilience, connectedness to the local community and organisational support [50-52]. Understanding the environment of rural practice prior to arrival in a rural community has been identified to influence retention. It is important that current and future rural physiotherapists are appropriately informed and prepared to prioritise service provision and make rationing decisions to meet service requirements within organisational constraints.

What do these findings mean physiotherapists working in regional, rural and remote areas? Firstly, the knowledge that some level of rationing was common across the cases and sites of this study may be a useful consideration when physiotherapists are required to ration services locally. The findings suggests the rationing or prioritising services forms part of rural physiotherapy practice. It is important therefore to provide education and strategies to assist physiotherapists working in regional, rural or remote practice to respond to situations where demand for services exceeds the available resources. There is an extensive literature on service planning and evaluation that provides comprehensive coverage to address issues relating to service prioritisation or rationing. The background information and findings of this study may provide some assistance in this area by posing the following recommendations for initial consideration.

Firstly, assess the current service demands in terms of key drivers. Consider, for instance, macro and meso level planning and policy, community demography and available physiotherapy services within the local community. Frame questions in terms of terms of technical and distributive criteria (Figure 1) by considering:

- The effectiveness and appropriateness of current service provision and the efficiency of the current service. For example, what are the organisational priorities that must be met? Has the effectiveness of services and interventions been maximised? Are there additional efficiency strategies that could be implemented?
- The local community need and the relative ability to benefit. What are the expectations of the local community? Are these expectations consistent with the organisations service priorities?

Secondly, assess the available physiotherapy capacity and capability of the service by considering workforce issues such as:

- the number of funded positions,
- the level of experience and skill mix
- the alignment of capability to service requirements
- vacancy rates, intention to stay and the odds of successful recruitment

Finally, assess the match of service demands to available resources. Where demand exceeds available resources and service effectiveness, efficiency and appropriateness have been optimised, then consider which of the rationing strategies to implement (Table 4).

**Figure 3:**
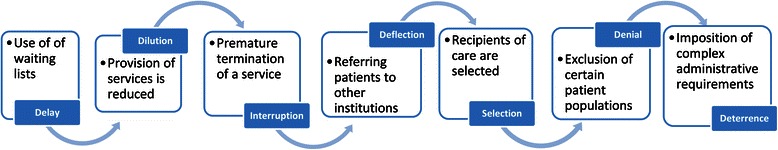
**A possible sequencing of rationing methods.**

- It may be possible to maintain the service scope, but manage the demand by using the rationing strategies of delay (waiting lists), dilution (decrease the frequency of treatment) or interruption (imposition of time limits).
- Where it is not possible to maintain the current service scope or respond to new service requests, consider firstly referral of clients to other services (deflection), then use the criteria described above to identify the recipients of care (selection) and clarify what will not be provided (denial). The adoption of deterrence strategies such as the imposition of complex administrative requirements is a less explicit approach (Figure 3).

### Limitations

This study was undertaken in one region within Australia which had a mixture of remote, rural and regional centres. Results may not be applicable to other areas with a different mixture of centres such as more remote locations and less regional centres. Although this study asked physiotherapists to identify decision-making stakeholders, not all stakeholders were involved as participants. This may have biased results, as for example state health department decision makers were not involved in this study and yet their decisions will influence the physiotherapy service provision in rural and regional areas. Also, the small number of consumer participants did not produce a detailed perspective from this key stakeholder group. Future studies could consider conducting local consumer focus groups. This study only investigated decision-making about physiotherapy services provision. The results may not be applicable to other health disciplines and there may be different factors and interactions in setting where there are interdisciplinary service delivery models. This can be seen by the differences seen in comparing public and private physiotherapy services decision-making.

## Conclusions

Deciding what health services are provided is a key consideration in delivering appropriate and accessible health care for rural populations. Participant perspectives revealed the impact of macro and meso level decisions on the capacity to provide physiotherapy services in the rural communities of this study. Increasing constraints meant that rationing of physiotherapy services, particularly within the public sector, was commonplace. The effective devolvement of rationing decisions to the micro level contributed to the stresses described by many participants working in public sector services. This study has revealed some consequences of service rationing that are relatively invisible at a system level yet so pertinent to individuals and communities. Decreased access to physiotherapy services was evident for example, for adults and children requiring neurological rehabilitation and for people requiring ongoing physiotherapy post-acute care. Responses of private physiotherapy providers indicate they are not positioned to address such service gaps, particularly when compounded by issues of affordability. Organisational and funding changes generated in recent state and national reforms have had significant, if unintended, consequences on the resources and capacity of physiotherapists in this study to deliver services in rural communities. This study provides insight into rural physiotherapy service provision not usually evident and can be used to inform health service planning and decision-making and education of current and future rural physiotherapists.

## Abbreviations

ABF: Activity based funding
APA: Australian Physiotherapy Association
ED: Emergency Department
FTE: Full time equivalent
ICU: Intensive care unit
